# Synchronization of a Non-Equilibrium Four-Dimensional Chaotic System Using a Disturbance-Observer-Based Adaptive Terminal Sliding Mode Control Method

**DOI:** 10.3390/e22030271

**Published:** 2020-02-27

**Authors:** Shaojie Wang, Amin Yousefpour, Abdullahi Yusuf, Hadi Jahanshahi, Raúl Alcaraz, Shaobo He, Jesus M. Munoz-Pacheco

**Affiliations:** 1 College of Electrical and Information Engineering, Shaoyang University, Shaoyang 422000, China; shaojiew@163.com; 2 School of Mechanical Engineering, College of Engineering, University of Tehran, Tehran 11155-4563, Iran; amin.yusefpour@ut.ac.ir; 3 Department of Computer Engineering, Biruni University, Istanbul 34010, Turkey; yusufabdullahi@fud.edu.ng; 4 Department of Mathematics, Federal University Dutse, Jigawa 7156, Nigeria; 5 Department of Mechanical Engineering, University of Manitoba, Winnipeg, MB R3T 5V6, Canada; Hadi.Jahanshahi@umanitoba.ca; 6 Research Group in Electronic, Biomedical and Telecommunication Engineering, University of Castilla-La Mancha, 16071 Cuenca, Spain; 7 School of Physics and Electronics, Central South University, Changsha 410083, China; heshaobo_123@163.com; 8 Faculty of Electronics Sciences, Benemerita Universidad Autonoma de Puebla, Mexico 72570, Mexico; jesusm.pacheco@correo.buap.mx

**Keywords:** four-dimensional chaotic systems, dynamical analysis, disturbance-observer, adaptive terminal sliding mode control, control input saturation, extended Kalman filter

## Abstract

In this paper, dynamical behavior and synchronization of a non-equilibrium four-dimensional chaotic system are studied. The system only includes one constant term and has hidden attractors. Some dynamical features of the governing system, such as invariance and symmetry, the existence of attractors and dissipativity, chaotic flow with a plane of equilibria, and offset boosting of the chaotic attractor, are stated and discussed and a new disturbance-observer-based adaptive terminal sliding mode control (ATSMC) method with input saturation is proposed for the control and synchronization of the chaotic system. To deal with unexpected noises, an extended Kalman filter (EKF) is implemented along with the designed controller. Through the concept of Lyapunov stability, the proposed control technique guarantees the finite time convergence of the uncertain system in the presence of disturbances and control input limits. Furthermore, to decrease the chattering phenomena, a genetic algorithm is used to optimize the controller parameters. Finally, numerical simulations are presented to demonstrate the performance of the designed control scheme in the presence of noise, disturbances, and control input saturation.

## 1. Introduction

Chaotic systems are currently attracting a considerable amount of attention thanks to their potential applications in a variety of fields [1,2,3,4,5,6]. Thus far, different chaotic systems have been introduced, including extreme multistable systems [7,8], multistable systems [9,10,11], and systems with multi-scroll attractors [12,13]. In addition, the first non-equilibrium chaotic flow was proposed by Sprott [14], and several non-equilibrium chaotic systems have been introduced and studied [15,16,17,18,19,20], because they present unanticipated responses to disturbances.

Some research studies have also proposed four-dimensional chaotic systems with special features. Among others, since Rössler studied the first four-dimensional chaotic system [21], a four-dimensional continuous-time autonomous no equilibria system with a cubic nonlinear term was proposed by Pham et al. [22]. A memristive system without any equilibrium was also presented by Bao et al. [23], who demonstrated that this system was able to exhibit chaotic, hyperchaotic, transient hyperchaotic, as well as periodic dynamics. Moreover, a four-dimensional chaotic system including nine terms and only one constant term, which either has a line of equilibria or does not possess equilibria, was very recently proposed by Zhang et al. [24].

In the last few years, a broad variety of techniques have also been proposed for controlling nonlinear and complex systems, including adaptive control, a backstepping approach, fuzzy control, optimal control, and sliding mode control [25,26,27,28,29,30,31,32,33,34]. In this regard, the control and synchronization of chaotic systems are also attracting a lot of attention [35,36,37,38]. For instance, Pérez-Cruz et al. proposed a novel linear feedback controller for synchronization of chaotic master and slave systems [39]. In another study, Pérez-Cruz also proposed an adaptive control scheme for synchronization of uncertain systems [40].

More studies on nonlinear controllers are still required, however, to improve their performance when dealing with some issues. To this end, most systems possess uncertain nonlinear dynamics in the presence of unknown external disturbances. In addition, the amount of control input must be considered due to the power consumption of actuators.

The present work addresses these aspects, and the contributions are presented as follows:

- The combination of disturbance-observer-based adaptive terminal sliding mode control (ATSMC) with a disturbance observer was developed for control and synchronization of an uncertain chaotic system in the presence of disturbances.
- The control input saturation was considered.
- An extended Kalman filter (EKF) approach was implemented with the controller for condition monitoring purposes. Indeed, this algorithm was used to estimate the actual amounts of the states of the system.
- A genetic algorithm (GA) optimization was used to reduce the chattering phenomena.
- Finally, numerical simulations illustrated the main characteristics and dynamical behaviors of the analyzed four-dimensional chaotic system, as well as the proposed controller for its synchronization.

The second section of the paper describes some characteristics of the proposed system, such as invariance and symmetry, the existence of attractors and dissipativity, chaotic flow with a plane of equilibria, and offset boosting of the chaotic attractor. This section also precisely details the proposed adaptive terminal sliding mode control (ATSMC) scheme, along with the designed extended Kalman filter (EKF) algorithm. The results of a simulation of the closed-loop system with incomplete state measurement and synchronization of the uncertain system are presented and discussed in the third section of the paper. Concluding remarks are summarized in the fourth section.

## 2. Methods

### 2.1. System Description and Dynamical Analysis

The governing equation of the analyzed four-dimensional chaotic system can be expressed as [41]:

(1) $$\dot{x}=y,\;\dot{y}=z,\;\dot{z}=w,\;\dot{w}=-aw+bx^{2}-cy^{2}+exy+fxz+g.$$

Note that the system is presented without control input, because only an analysis of its dynamical behavior is pursued in this section. The governing system is said to be invariant under the transformation (x,y,z,w)→(−x,−y,−z,w), an approximately $180^{{}^{\circ}}$ rotation about the w-axis. That is to say, on reflection in the w-axis and, for all of the values of the parameters in the system, this symmetry remains.

Given the dynamical system under consideration reported in Equation (1), its volume contraction rate can be expressed in terms of the following Lie derivative:

(2) $$\Delta V=\frac{\partial \dot{x}}{\partial x}+\frac{\partial \dot{y}}{\partial y}+\frac{\partial \dot{z}}{\partial z}+\frac{\partial \dot{w}}{\partial w}=-a.$$

The system presented by Equation (1) is a dissipative one, and its exponential contraction can be expressed by:

(3) $$\frac{dV}{dt}=e^{-a}.$$

This shows that a volume element $V_{0}$ is apparently contracted by the flow into a volume element $V_{0}\times e^{-at}$ in time t. Hence, each volume containing the trajectory of this dynamical system shrinks to zero whenever t→∞ at an exponential rate −a. This way, all these orbits are eventually confined to a specific subset that has zero volume, and the asymptotic motion settles onto an attractor of (1).

Taking Equation (1) into consideration with a,b,c,d,e,f, and g representing parameters, the chaotic flow with a plane of equilibria has all of the xw-plane as the points of the equilibrium (F*) in the sense that z=y=0. From Equation (2), it can be concluded that the divergence is negative for a>1. Therefore, the dissipativity condition with regard to the existence of attractive sets in the system is held for a>1. The Jacobian matrix and its value at F* are stated as:

(4) $$J\left(x,y,z,w\right)=\left(\begin{array}{ccc} 0 & \begin{array}{cc} 1 & 0 \end{array} & 0\\ \begin{array}{c} 0\\ 0 \end{array} & \begin{array}{cc} 0 & 1\\ 0 & 0 \end{array} & \begin{array}{c} 0\\ 1 \end{array}\\ ey+fz & \begin{array}{cc} ex & fx \end{array} & -a \end{array}\right),$$

(5) $$J\left(F*\right)=\left(\begin{array}{ccc} 0 & \begin{array}{cc} 1 & 0 \end{array} & 0\\ \begin{array}{c} 0\\ 0 \end{array} & \begin{array}{cc} 0 & 1\\ 0 & 0 \end{array} & \begin{array}{c} 0\\ 1 \end{array}\\ 0 & \begin{array}{cc} x & x \end{array} & -a \end{array}\right).$$

The eigenvalues for the system in F* are computed and the following are obtained:

(6) $$0,\frac{1}{6}{\left(-8a^{3}-36xa+108x+12\sqrt{-12a^{3}x-3a^{2}x^{2}-54ax^{2}-12x^{3}+81x^{2}}\right)}^{\frac{1}{3}}\;-\frac{6\left(-\frac{1}{3}x-\frac{1}{9}a^{2}\right)}{{\left(-8a^{3}-36xa+108x+12\sqrt{-12a^{3}x-3a^{2}x^{2}-54ax^{2}-12x^{3}+81x^{2}}\right)}^{\frac{1}{3}}}-\frac{1}{3}a.$$

**Figure 1:**
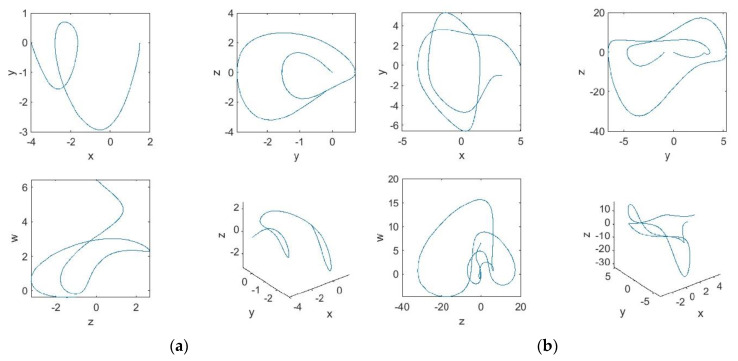
Projections in the stated planes with a suitable choice of parameter values. (**a**) xy, yz, wz, and xyz planes with initial conditions [x(0),y(0),z(0),w(0)]=[1.5,0,−1,−0.3]. (**b**) xy, yz, wz, and xyz planes with initial conditions [x(0),y(0),z(0),w(0)]=[3.5,−1,0,0.5].

For the choice of parameters b=1, a=−1, c=2, e=f=−1, and g=2, and the initial conditions [x(0),y(0),z(0),w(0)]=[1.5,0,−1,−0.3] and [x(0),y(0),z(0),w(0)]=[3.5,−1,0,0.5], the plots for the state space are depicted through two-dimensional and three-dimensional representations, such as can be seen in Figure 1.

The distinguishing device property (1) is a one-constant offset boost. From a physical point of view, the ability of amplitude control is a relevant feature of a potential chaos generator [42,43,44,45]. The added feedback status can be seen as a valuable replacement for controlling the variables’ amplitude. In Equation (1), the state variable x only exists in the fourth line, so x is boostable offset. In other words, the transformation x→x+k will balance the state variable x, being k a constant. Thus, Equation (1) can be expressed as:

(7) $$\frac{dx}{dt}=y\;\frac{dy}{dt}=z\;\frac{dz}{dt}=w\;\frac{dw}{dt}=-aw+b{\left(x+k\right)}^{2}-cy^{2}+e\left(x+k\right)y+f\left(x+k\right)z+g.$$

A novel sliding-mode-based control algorithm is implemented in the following section to synchronize the chaotic behavior of the proposed system.

### 2.2. Controller Design

By defining $x_{1}=x\;,\;x_{2}=y,\;x_{3}=z,\;\mathrm{and}\;x_{4}=w,\;$the state space of a nonlinear system with a control input in the presence of the perturbance can be expressed as follows:

(8) $$\left\{ \begin{array}{ll} {\dot{x}}_{i}=f_{i}\left(x\right) & \;i=1,2,3\\ {\dot{x}}_{4}=f_{4}\left(x\right)+\Delta f(x)+u+d(t), & \end{array}\right.$$

where $f_{i}\left(x\right)=x_{i+1}\;$for i=1, 2, 3 and$\;f_{4}\left(x\right)=-ax_{4}+bx_{1}^{2}-cx_{2}^{2}+ex_{1}x_{2}+fx_{1}x_{3}+g\;$. Δf is the uncertainty of the system. The external disturbance and the control input are represented by d(t) and u, respectively.

#### 2.2.1. Fast Disturbance Observer

Because of physical limitations, input saturation is a prevalent phenomenon in many real systems. Thus, in the present study, the existence of input saturation is considered in the control design procedure. By considering these constraints, the control input u is given by:

(9) $$u=\{\begin{array}{c} u_{max}\;\;\;\;\;\;\;\;\;\;\;\;\;\;\;\;\;\;\;\;\;\;\;\;\;if\;u_{d}>u_{max},\;\;\;\\ u_{d\;\;\;\;\;\;\;\;\;\;\;\;\;\;\;\;\;\;\;}\;\;\;\;\;\;if\;\;u_{min}<u_{d}<u_{max,}\\ u_{min}\;\;\;\;\;\;\;\;\;\;\;\;\;\;\;\;\;\;\;\;\;\;\;\;\;if\;u_{d}<u_{min},\;\;\;\;\;\; \end{array}$$

where $u_{min}$ and $u_{max}$ represent the limits for the control input saturation and the designed control $u_{d}\;$will be obtained later. Substituting $\tilde{u}=u-u_{d}$ in Equation (8) yields:

(10) $$\begin{array}{ll} {\dot{x}}_{i}=f_{i}\left(x\right) & \;i=1,2,3,\\ {\dot{x}}_{4}=f_{4}\left(x\right)+\left(u_{d}+\tilde{u}\right)+d\left(t\right)\;=f_{4}\left(x\right)+u_{d}+D, & \end{array}$$

where $D=\tilde{u}+d$ is a compound disturbance that the system is exposed to in the presence of input saturation, external perturbation, and dynamic uncertainties.

**Assumption** **1.** *The compound disturbance* (D) *is bounded, i.e.,* β>|D| *, and* β *is a positive parameter.*

Now, the disturbance for the unknown compound disturbance is given by [46]:

(11) $$\hat{D}=-ks_{d}-\beta \mathrm{sign}\left(s_{d}\right)-\varepsilon s^{\frac{p_{0}}{q_{0}}}-\left|f\left(x\right)\right|\mathrm{sign}\left(s_{d}\right)-f\left(x\right).$$

The auxiliary variable $s_{d}$ is given by:

(12) $$s_{d}=z_{do}-x_{4},$$

(13) $${\dot{z}}_{do}=-ks_{d}-\beta \mathrm{sign}\left(s_{d}\right)-\varepsilon s^{\frac{p_{0}}{q_{0}}}-\left|f\left(x\right)\right|\mathrm{sign}\left(s_{d}\right)+u_{d}$$

where k and ε are positive parameters. In addition, $p_{0}$ and$\;q_{0}$ are odd positive integers with $p_{0}<q_{0}$. By considering Equations (10), (12) and (13), the following can be obtained:

(14) $${\dot{s}}_{d}={\dot{z}}_{do}-{\dot{x}}_{4}=-ks_{d}-\beta \mathrm{sign}\left(s_{d}\right)-\varepsilon s^{\frac{p_{0}}{q_{0}}}-\left|f\left(x\right)\right|\mathrm{sign}\left(s_{d}\right)-f\left(x\right)-D.$$

Then, by considering Equations (10), (11) and (12), it can be seen that:

(15) $$\tilde{D}=\hat{D}-D=-ks_{d}-\beta \mathrm{sign}\left(s_{d}\right)-\varepsilon s^{\frac{p_{0}}{q_{0}}}-\left|f\left(x\right)\right|\mathrm{sign}\left(s\right)-f\left(x\right)-D\;=-ks_{d}-\beta \mathrm{sign}\left(s_{d}\right)-\varepsilon s_{d}{}^{\frac{p_{0}}{q_{0}}}-\left|f\left(x\right)\right|\mathrm{sign}\left(s_{d}\right)-f\left(x\right)-{\dot{x}}_{4}+f\left(x\right)+u_{d}\;=-ks_{d}-\beta \mathrm{sign}\left(s_{d}\right)-\varepsilon s_{d}{}^{\frac{p_{0}}{q_{0}}}-\left|f\left(x\right)\right|\mathrm{sign}\left(s_{d}\right)+u_{d}-{\dot{x}}_{4}={\dot{z}}_{od}-{\dot{x}}_{4}={\dot{s}}_{d}.$$

To show the stability, as well as assess the tracking of the disturbance observer in infinite time, Theorem 1 and Lemma 1 were used.

**Lemma** **1.** *Consider the continuous positive definite function* V(t) *that meets the following inequalities [47]:*

(16) $$\dot{V}\left(t\right)+\vartheta V\left(t\right)+\xi V^{\chi }\leq 0,\;\forall t>t_{0}.$$

As a result, V(t) converges to the equilibrium point in the finite time $t_{s}$ as:

(17) $$t_{s}\leq t_{0}+\frac{1}{\vartheta \left(1-\chi \right)}\ln\frac{\vartheta V^{1-\chi }\left(t_{0}\right)+\xi }{\xi },$$

where parameters 0<χ<1 and ϑ> ξ >0.

**Theorem** **1.** *For the uncertain system introduced by Equation (10), the disturbance approximation error computed as Equation (15) converges to zero in a finite time by applying the disturbance observer described in Equations (11)–(13).*

**Proof.**  Let a Lyapunov function candidate be:

(18) $$V_{0}=\frac{1}{2}s_{d}{}^{2}.$$

The first-time derivative of $V_{0}$ is:

(19) $${\dot{V}}_{0}=s_{d}{\dot{s}}_{d}=s_{d}\left(-ks_{d}-\beta \mathrm{sign}\left(s_{d}\right)-\varepsilon s_{d}{}^{\frac{p_{0}}{q_{0}}}-\left|f\left(x\right)\right|s\mathrm{ign}\left(s_{d}\right)-f\left(x\right)-D\right)\;\leq -ks_{d}{}^{2}-\beta s_{d}\mathrm{sign}\left(s_{d}\right)-\varepsilon s_{d}{}^{\frac{p_{0+}q_{0}}{q_{0}}}-\left|f\left(x\right)\right|s_{d}\;\mathrm{sign}\left(s_{d}\right)-s_{d}f\left(x\right)-s_{d}D\;\leq -ks_{d}{}^{2}-\beta \left|s_{d}\right|-\varepsilon s_{d}{}^{\frac{p_{0+}q_{0}}{q_{0}}}-\left|f\left(x\right)\right|\left|s_{d}\right|-s_{d}f\left(x\right)+\left|s_{d}\right|\left|D\right|\;\leq -ks_{d}{}^{2}-\varepsilon s_{d}{}^{\frac{p_{0+}q_{0}}{q_{0}}}\leq -2kV_{0}-2^{(p_{0}+q_{0})/2q_{0}}\varepsilon V_{0}^{(p_{0}+q_{0})/2q_{0}}.$$

**Remark** **1.** *Based on Theorem 1 and Lemma 1, in a finite time the disturbance approximation error converges to zero. The convergence time of the disturbance estimator is also given by:*

(20) $$t_{s}<t_{0}+\frac{q_{0}}{k\left(p_{0}+3q_{0}\right)}\ln\left(\frac{ks_{0}^{(q_{0}-p_{0})/q_{0}}t_{0}}{\varepsilon }+1\right),$$

in which $t_{0}$ indicates the initial time.

□

#### 2.2.2. Adaptive Sliding Mode Control

Tracking control with the adaptive terminal sliding mode technique is developed here for the case where all states of the system are available. The tracking error of the system can be expressed as:

(21) $$e_{i}=x_{i}-x_{di}\;$$

$x_{di}\;\mathrm{being}\;$the desired value of state $x_{i}$. To develop ATSMC, the sliding mode function can be defined as:

(22) $$S\left(t\right)=e_{4}+c_{3}e_{3}+c_{2}e_{2}+c_{1}e_{1},$$

where $c_{1},c_{2},c_{3}$ are the design parameters and should be chosen as positive constants for which the polynomial $s^{n-1}+c_{n-1}s^{n-2}+\cdot \cdot \cdot +c_{1}$ is Hurwitz. As a novel approach, an adaptive terminal sliding mode tracking control method with a fast disturbance observer is proposed. The adaptive surface is assumed to be:

(23) $$s_{n}\left(t\right)=s\left(t\right)+\hat{\alpha }s\left(t\right)+s_{d}\left(t\right),$$

and then the proposed disturbance-observer-based adaptive terminal sliding control technique is designed as:

(24) $$u_{d}=-\left(c_{3}e_{4}+c_{2}e_{3}+c_{1}e_{2}+f_{4}\left(x\right)-{\dot{x}}_{d4}+\hat{\alpha }\dot{s}+\delta s_{n}+\zeta s_{n}^{\frac{p}{q}}+\hat{D}\right),$$

where ζ and δ are positive design parameters. In addition, p and q are odd positive integers, where p≤q and $\hat{\alpha }\;$is an adjustable parameter that will be updated using the following update law:

(25) $$\dot{\hat{\alpha }}=-\eta_{1}ss_{n},$$

where $\eta_{1}\;$is a positive parameter.

**Theorem** **2.** *By considering Equation (24) and supposing that state information is fully available, the tracking error of the uncertain nonlinear system described in Equation (8) converges to zero in a finite time based on the proposed fast disturbance-observer-based ATSMC technique.*

**Proof.**  Choosing the Lyapunov function candidate as:

(26) $$V\left(s_{n}\right)=\frac{1}{2}s_{n}^{2},$$

its time derivative is given by:

(27) $$\dot{V}\left(s_{n}\right)=s_{n}{\dot{s}}_{n}.$$

Considering Equations (22) and (23), it can then be obtained that:

(28) $$\dot{V}=s_{n}\left(\dot{s}+\dot{\hat{\alpha }}s+\hat{\alpha }\dot{s}+{\dot{s}}_{d}\right)=s_{n}\left(c_{1}\;{\dot{e}}_{1}+c_{2}{\dot{e}}_{2}+c_{3}{\dot{e}}_{3}+{\dot{e}}_{4}+\dot{\hat{\alpha }}s+\hat{\alpha }\dot{s}+{\dot{s}}_{d}\right)\;=s_{n}\left(c_{1}\;{\dot{e}}_{1}+c_{2}{\dot{e}}_{2}+c_{3}{\dot{e}}_{3}+{\dot{x}}_{4}-{\dot{x}}_{d4}+\dot{\hat{\alpha }}s+\hat{\alpha }\dot{s}+{\dot{s}}_{d}\right).$$

According to Equation (8), this expression can be rewritten as:

(29) $$\dot{V}=s_{n}\left(c_{1}\;{\dot{e}}_{1}+c_{2}{\dot{e}}_{2}+c_{3}{\dot{e}}_{3}+f_{4}\;\left(x\right)+u_{d}+D\left(t\right)-{\dot{x}}_{d4}+\dot{\hat{\alpha }}s+\hat{\alpha }\dot{s}+{\dot{s}}_{d}\right).$$

Substituting the control law described by Equation (24) into Equation (29) results in:

(30) $$\dot{V}=s_{n}\left(c_{1}\;{\dot{e}}_{1}+c_{2}{\dot{e}}_{2}+c_{3}{\dot{e}}_{3}-\left(c_{3}e_{4}+c_{2}e_{3}+c_{1}e_{2}\right)+D\left(t\right)-\hat{D}-\delta s_{n}-\zeta s_{n}^{\frac{p}{q}}+\dot{\hat{\alpha }}s+{\dot{s}}_{d}\right).$$

According to Equation (8), it is known that for =1,2,3, ${\dot{e}}_{i}=e_{i+1}$; therefore:

(31) $$\dot{V}=s_{n}\left(D\left(t\right)-\hat{D}-\delta s_{n}-\zeta s_{n}^{\frac{p}{q}}+\dot{\hat{\alpha }}s+{\dot{s}}_{d}\right),\;$$

and considering Equation (15), $D-\hat{D}=-{\dot{s}}_{d}$, it is then obtained that:

(32) $$\dot{V}=s_{n}\left(\dot{\hat{\alpha }}s-\delta s_{n}-\zeta s_{n}^{\frac{p}{q}}\right)\;=s_{n}\left(-\eta_{1}s^{2}s_{n}-\delta s_{n}-\zeta s_{n}^{\frac{p}{q}}\right)\leq -\delta s_{n}^{2}-\zeta s_{n}^{\frac{p+q}{q}}\leq -2\delta V-2^{\frac{p+q}{2q}}\zeta V^{\frac{p+q}{2q}}\;.$$

**Remark** **2.** Considering Equation (32) and Lemma 1, it can be confirmed that the resulting adaptive terminal sliding mode tracking control technique satisfies the Lyapunov condition; in a finite time, the trajectories of the system converge to the desired path.

□

#### 2.2.3. Extended Kalman Filter

It is known that the ATSMC technique requires the states of the system, and thus the acutal amounts being used for the controller. To estimate the state vector of the system, the extended Kalman filter (EKF) was used in this study. This algorithm provides a solution for a nonlinear system that directly deals with the effects of the disturbance noises, including measurement and system noises. Additionally, by using the EKF, the errors in the parameters will be handled as noise. Uncertainties, such as parameter mismatches and noises, may ruin the chaos control.

The EKF is added to the control scheme system; hence, the controller’s accuracy can be significantly improved in the presence of the noises. The discrete dynamic state model for the system is as follows:

(33) $$x\left(k\right)=f\left(x_{k-1}\right)+r_{k-1},\;y\left(k\right)=h\left(x_{k-1}\right)+v_{k-1},$$

where r and v are the process and measurement noise vectors, respectively, $x=\left[x_{1},x_{2},x_{3},\;x_{4}\right]\;$is the state vector, and y is the output of the system. The EKF algorithm can be given by the following recursive equation. The first step (prediction) provides a prediction of the states and the covariance matrix based on previous estimates, i.e.,

(34) $${\hat{x}}_{\left(k|k-1\right)}=f\left({\hat{x}}_{\left(k-1|k-1\right)}\right),$$

(35) $$P_{\left(k|k-1\right)}=F_{\left(k-1\right)}P_{\left(k-1|k-1\right)}F_{\left(k-1\right)}^{T}+Q_{\left(k-1\right)},$$

$\mathrm{where}\;{\hat{x}}_{\left(k|k-1\right)}$ is the estimated state at time k using data from time 0 to time k, f is the state transition function, and $P_{\left(k|k-1\right)}$ denotes the prediction error covariance matrix. The second step corrects the covariance matrix and predicted states, which is realized by the following recursive relations:

(36) $$B_{\left(K\right)}=q_{k}-h\left({\hat{x}}_{\left(k|k-1\right)}\right),$$

(37) $$S_{\left(K\right)}=H_{\left(k-1\right)}P_{\left(k|k-1\right)}H_{\left(k-1\right)}^{T}+R_{k},$$

(38) $$K_{\left(K\right)}=P_{\left(k|k-1\right)}H_{\left(k\right)}^{T}S_{\left(K\right)}^{-1},$$

(39) $${\hat{x}}_{\left(k|k\right)}={\hat{x}}_{\left(k|k-1\right)}+K_{\left(K\right)}B_{\left(K\right)},$$

(40) $$P_{\left(k|k\right)}=P_{\left(k|k-1\right)}-K_{\left(K\right)}S_{\left(K\right)}K_{\left(K\right)}^{T},$$

K,B,S being the estimation gains, R and Q the covariances of measurements and process noises, and F and H the Jacobian matrix of the system, given by:

(41) $${\left[F_{k}\right]}_{i,j}={\frac{\partial f_{i}\left(X\right)}{\partial x_{j}}|}_{x={\hat{x}}_{\left(k|k\right)}}\;,$$

(42) $${\left[H_{k}\right]}_{i,j}={\frac{\partial h_{i}\left(X\right)}{\partial x_{j}}|}_{x={\hat{x}}_{\left(k|k\right)}}.$$

**Figure 2:**
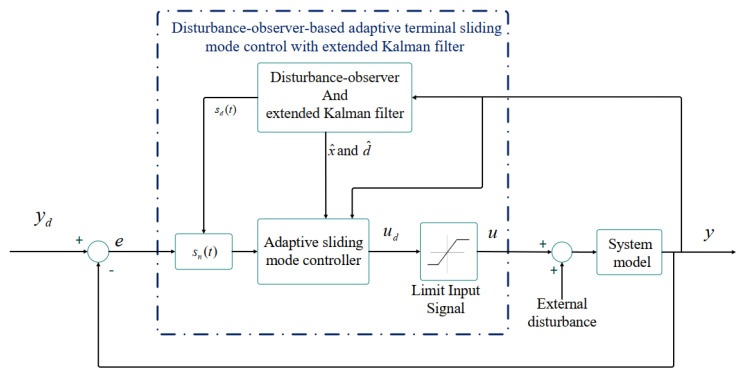
A block diagram describing the proposed disturbance-observer-based adaptive terminal sliding mode control (ATSMC) technique with the extended Kalman filter (EKF) algorithm.

As an summary, Figure 2 illustrates the procedure of the obtained control scheme. The disturbance-observer-based ATSMC technique with EKF has been designed for the control and synchronization of uncertain nonlinear systems. Clearly, the states of the system, which are estimated through the EKF algorithm, are necessary for ATSMC. The control input saturation also affects the system, and therefore this issue was considered for its control. Actually, a limitation operator was introduced in Equation (9) and, in this condition, the stability and convergence of the closed-loop system were proven. Consequently, even when there exists input saturation, the system will reach its desired value because disturbances are bounded.

## 3. Results and Discussion

In this section, a numerical simulation of the proposed control method is introduced and discussed. The equation of the system with the control input and external disturbance can be expressed as:

(43) $$\dot{x}=y,\;\dot{y}=z,\;\dot{z}=w,\;\dot{w}=-aw+bx^{2}-cy^{2}+exy+fxz+g+u+d\left(t\right),$$

where u is the control input and the external disturbance d(t) is given by 0.1sin(0.1πt).

GA optimization has been combined with the proposed control technique to reduce the chattering phenomena. In fact, using this approach, the parameters of the control scheme can be determined. The cost function of the GA was considered to be:

(44) $$J=min\sum \eta_{1}e^{T}e+\eta_{2}{\dot{e}}^{T}\dot{e},$$

where$\;e\left(t\right)=\left[e_{x},\;e_{y},\;e_{z},\;e_{w}\right]={\left[x-x_{d},y-y_{d},z-z_{d},w-w_{d}\right]}^{T}$ and the weights of the cost function were established as $\eta_{1}=1$ and $\eta_{2}=5$. The term $\eta_{2}{\dot{e}}^{T}\dot{e}$ was specifically included to decrease the chattering in the response of the system and the parameters $c_{1},\;c_{2},\;c_{3}$, $\eta_{1}$, ζ, δ, ε, and k were obtained to minimize the cost function. The population size was considered to be 250, the number of generations 200, the mutation probability 0.05, and the crossover probability 0.9. The rest of design parameters were:

(45) $$p_{0}=1,\;\;q_{0}=11,\;p_{0}=3,\;\;q_{0}=7,\;\mathrm{and}\;\beta =500.$$

### 3.1. Chaos Control with Incomplete State Measurement

In this part, it is assumed that the value of state x is unavailable. According to this uncertain state, the EKF observer was used to estimate x. The estimated value $x_{e}$ was used for the control scheme. The initial conditions were considered to be [0,−1, 0,−1.5]. The measurement and process noises were established as white noise with a zero mean value and the following covariance matrices R and Q, respectively:

(46) $$\;R\left[\begin{array}{ccc} 0.1 & 0 & 0\\ 0 & 0.1 & 0\\ 0 & 0 & 0.1 \end{array}\right],\;\;Q=\left[\begin{array}{cccc} 0.1 & 0 & 0 & 0\\ 0 & 0.1 & 0 & 0\\ 0 & 0 & 0.1 & 0\\ 0 & 0 & 0 & 0.1 \end{array}\right].$$

**Figure 3:**
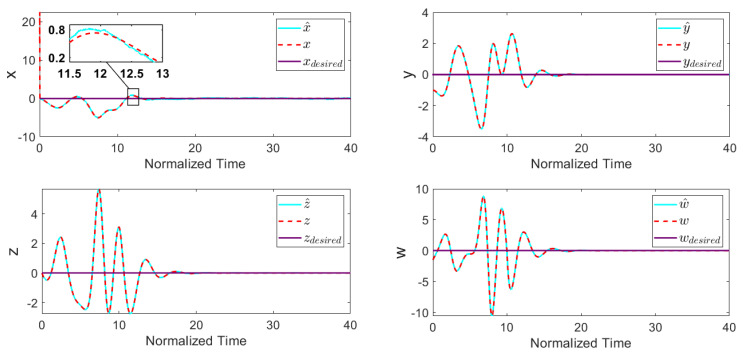
System states with disturbance-observer-based ATSMC with EKF (*Tstart* = 10).

**Figure 4:**
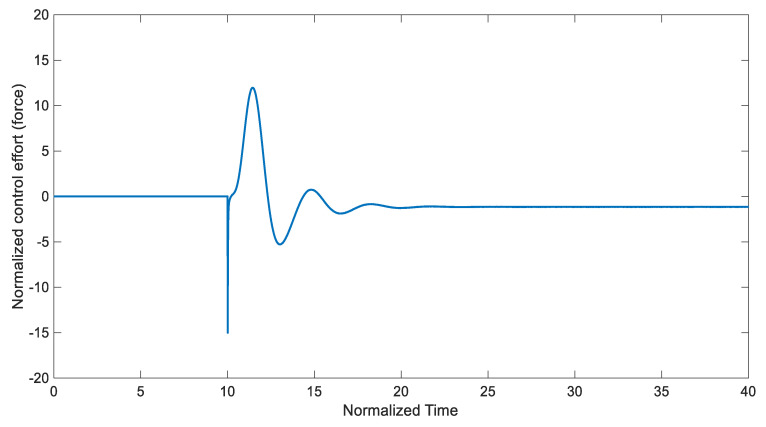
Time history of the control input for the proposed control scheme (*Tstart* = 10).

The feasible control input saturation was also considered by setting $u_{\max}=15$ and $u_{\min}=-15$. The performance and effectiveness of the proposed control method were investigated by obtaining the time-response of the system. For this purpose, the controller was turned on at *Tstart* = 10. Figure 3 depicts the stabilized states of the system, where in a short time period they converged to the desired values. From Figure 3, it can be concluded that the EKF predicts state x correctly and improves the controller’s behavior significantly. Figure 4 shows the control input signal based on the applied control scheme.

Table 1 presents the designed control input and settling time ($T_{s}$) values for the system, where ${\left||\;|\right|}_{2}\;$and ${\left||\;|\right|}_{\infty }$ indicate the Euclidian norm and the infinity norm, respectively. All these simulation outcomes (Figure 3 and Figure 4 and Table 1) show that the presented uncertain nonlinear system was stabilized in the existence of noises and time-varying external disturbances. Hence, the aforementioned controller satisfied the expected performance.

### 3.2. Adaptive Synchronization of the Uncertain Chaotic System

The disturbance-observer-based ATSMC with EKF was used to synchronize the chaotic system. The case with little knowledge of the slave system’s parameters will be investigated, and in this way the robustness of the proposed method will be proven. The slave system is taken from Equation (43), and the master system is as follows:

(47) $${\dot{x}}_{m}=y_{m},\;{\dot{y\;}}_{m}=z_{m},\;{\dot{z}}_{m}=w_{m},\;{\dot{w}}_{m}=-a_{m}w_{m}+b_{m}x_{m}^{2}-c_{m}y_{m}^{2}+e_{m}x_{m}y_{m}+f_{m}x_{m}z_{m}+g_{m}.$$

The master system’s parameters were $a_{m}=1.05$, $b_{m}=0.7$, $c_{m}=0.19$, $e_{m}=1.37$, $f_{m}=1.79$, and $g_{m}=-4$, and the initial conditions were [−1, −1, −1, −1]. The case with little knowledge of the slave system’s parameters was considered by setting them to 70% of their actual value, i.e.,

(48) $$\hat{a}=0.7\times a,\hat{b}=0.7\times b,\hat{c}=0.7\times c,\hat{e}=0.7\times e,\hat{g}=0.7\times g.$$

The incorrect parameters $\hat{a},\;\hat{b},\;\hat{c},\;\hat{e},\hat{f}$, and $\hat{g}$ were then used for the controller, instead of the correct values a, b, c, e, f, and g. It was also assumed that the value of state x from the slave system is unavailable.

**Figure 5:**
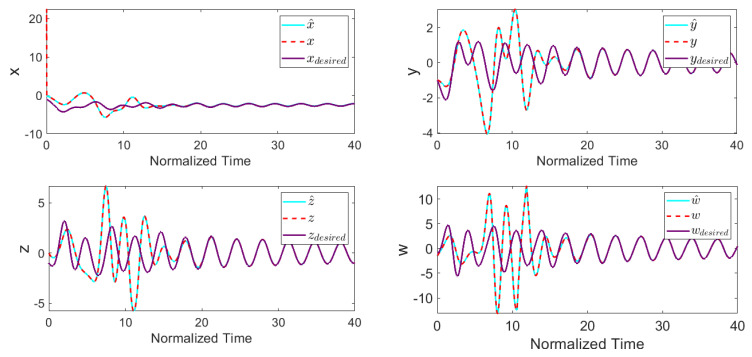
Synchronization results for the chaotic system using disturbance-observer-based ATSMC with the EKF (*Tstart* = 10).

**Figure 6:**
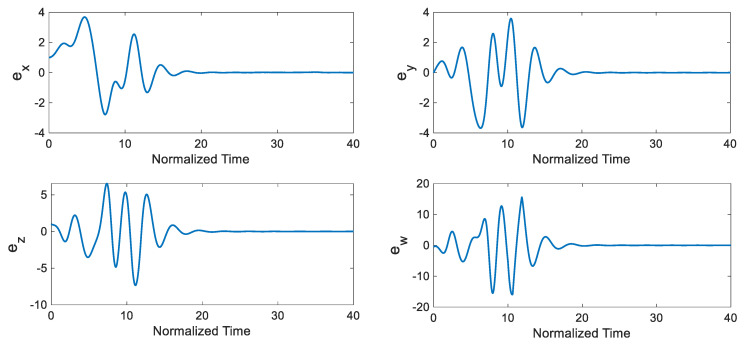
Synchronization errors in the chaotic system using disturbance-observer-based ATSMC with the EKF (*Tstart* = 10).

**Figure 7:**
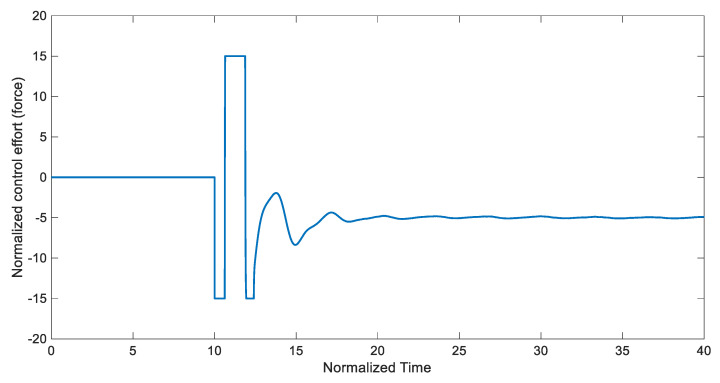
Control input for synchronization of the chaotic system using disturbance-observer-based ATSMC with the EKF (*Tstart* = 10).

Under these conditions, synchronization time trajectories and synchronization errors are presented in Figure 5 and Figure 6, respectively. The control input’s evolution over time is illustrated in Figure 7. Note that one of the most significant advantages of the developed control scheme is tracking control when the control input is saturated. That situatation is especially relevant in practical applications. The Lyapunov stability theorem of the designed controller ensures that the closed-loop system is stable while there are control input limits.

Hence, the described simulation outcomes indicate that the designed control scheme is capable of synchronizing chaotic systems in the presence of dynamic uncertainties, process and measurement noise, external perturbations, and control input saturation.

## 4. Conclusions

A non-equilibrium four-dimensional chaotic system with specific features was studied. Some of the dynamical characteristics of the system, including invariance and symmetry, the existence of attractors and dissipativity, chaotic flow with a plane of equilibria, and offset boosting of the chaotic attractor, were discussed. A disturbance-observer-based ATSMC scheme was designed for the control and synchronization of the chaotic system in the presence of dynamic uncertainties, external disturbances, and control input saturation. To guarantee the performance of the proposed control scheme in the presence of noises and uncertainties, the EKF algorithm was used. Additionally, a genetic algorithm was used to optimize the controller parameters, thus reducing the chattering phenomena. Finally, some simulation results were presented to exhibit the performance of the suggested control method for uncertain chaotic systems in the presence of noise and disturbances. In future work, the practical application of such a system will be analyzed. For instance, given its chaotic dynamics, the system could be useful for the development of chaos-based applications. Moreover, the extension of the proposed control method could also be used for fractional-order systems.

## Figures and Tables

**Table 1:** Norms of control input and values of settling time (T_s) based on the proposed control scheme.

${\left||u|\right|}_{2}$	${\left||u|\right|}_{\infty }$	$T_{s}\left(x\right)$	$T_{s}\left(y\right)$	$T_{s}\left(z\right)$	$T_{s}\left(w\right)$
212.5013	15	17.7775	17.0458	16.2175	16.7562
